# Endogenous Multiple Exon Skipping and Back-Splicing at the *DMD* Mutation Hotspot

**DOI:** 10.3390/ijms17101722

**Published:** 2016-10-13

**Authors:** Hitoshi Suzuki, Yoshitsugu Aoki, Toshiki Kameyama, Takashi Saito, Satoru Masuda, Jun Tanihata, Tetsuya Nagata, Akila Mayeda, Shin’ichi Takeda, Toshifumi Tsukahara

**Affiliations:** 1 School of Materials Science, Japan Advanced Institute of Science and Technology, Nomi, Ishikawa 923-1292, Japan; tukahara@jaist.ac.jp; 2 Department of Molecular Therapy, National Institute of Neuroscience, National Center of Neurology and Psychiatry (NCNP), Kodaira, Tokyo 187-8502, Japan; tsugu56@ncnp.go.jp (Y.A.); tksaito@ncnp.go.jp (T.S.); masuda@ncnp.go.jp (S.M.); tanihata@ncnp.go.jp (J.T.); t-naga.nuro@tmd.ac.jp (T.N.); takeda@ncnp.go.jp (S.T.); 3 Division of Gene Expression Mechanism, Institute for Comprehensive Medical Science, Fujita Health University, Toyoake, Aichi 470-1192, Japan; tkame@fujita-hu.ac.jp (T.K.); mayeda@fujita-hu.ac.jp (A.M.); 4 Graduate School of Medical and Dental Sciences, Tokyo Medical and Dental University, Bunkyoku, Tokyo 113-0034, Japan

**Keywords:** pre-mRNA splicing, *DMD*, circular RNA, multiple exon skipping

## Abstract

Duchenne muscular dystrophy (DMD) is a severe muscular disorder. It was reported that multiple exon skipping (MES), targeting exon 45–55 of the *DMD* gene, might improve patients’ symptoms because patients who have a genomic deletion of all these exons showed very mild symptoms. Thus, exon 45–55 skipping treatments for DMD have been proposed as a potential clinical cure. Herein, we detected the expression of endogenous exons 44–56 connected mRNA transcript of the *DMD* using total RNAs derived from human normal skeletal muscle by reverse transcription polymerase chain reaction (RT-PCR), and identified a total of eight types of MES products around the hotspot. Surprisingly, the 5′ splice sites of recently reported post-transcriptional introns (remaining introns after co-transcriptional splicing) act as splicing donor sites for MESs. We also tested exon combinations to generate *DMD* circular RNAs (circRNAs) and determined the preferential splice sites of back-splicing, which are involved not only in circRNA generation, but also in MESs. Our results fit the current circRNA-generation model, suggesting that upstream post-transcriptional introns trigger MES and generate circRNA because its existence is critical for the intra-intronic interaction or for extremely distal splicing.

## 1. Introduction

Duchenne muscular dystrophy (DMD), which involves a progressive deterioration of muscle function [1], is caused by frame-shifting deletions or nonsense mutations in the *DMD* gene [2]. Becker muscular dystrophy (BMD) has milder symptoms than DMD and is mostly caused by in-frame deletions in the *DMD* gene, which is able to express an internally truncated, but partially functional, protein [3]. In recent years, exon-skipping therapies targeting a single exon that artificially induce target splicing and in-frame mRNA to restore the dystrophin protein have been developed as promising therapeutic approaches; however, these approaches are highly mutation-specific and personalized [4]. To overcome this disadvantage of single exon-skipping therapy, the induction of multiple exon skipping (MES) was proposed as a future therapy. There are two regions of the deletion mutation “hotspot” (exon 3–9, exon 45–55) in the *DMD* gene. We focused the main deletion mutation “hotspot” between exon 45 and exon 55 because this represented up to 63% of patients with the deletion mutations [5]. Symptoms of patients who have deletions of exons 45–55 are clearly related to remarkably mild muscle weakness; some of them are almost asymptomatic. In spite of large deletions, these patients are BMD patients having an in-frame mutation [6]. Therefore, a novel therapy based on the induction of the MES from exons 45 to 55 has been tested in mice and patient-derived cells [7].

Circular RNAs (circRNAs) were characterized recently as a group of physiological RNA molecules in eukaryotes [8,9]. Thousands of circRNAs are expressed widely and substantially in eukaryotic cells and tissues. The physiological functions of most circRNA species have not been clarified, with a few exceptions. For example, *CDR1as* (complementarity-determining region 1 antisense, or ciRS-7) circRNA functions as a sponge for microRNA-7 (miR-7) [10,11]. It was reported that EIcircRNAs (exon–intron circular RNA), which had a retaining intron between exons, could regulate the transcription of each parental gene [12].

It has been suggested that exonic circRNAs form a circular structure via splicing reactions [13,14,15]. Exonic circRNAs have a unique scrambled sequence that results in back-splicing (so-called head-to-tail splicing or circle splicing), which consists of a sequence comprising the 3′ end of the downstream exon connected to the 5′ end of the upstream exon (or the same downstream exon) [8,16]. It has been hypothesized that a by-product of MES forms a large lariat containing skipped exons and acts as a circRNA precursor during back-splicing [9,10,11,12,13,14,15,16]. The supportive data that circRNA could be generated through the exon-containing lariat precursor was shown in yeast [17]. However, the results of deep-sequencing projects in humans and mice indicated that complementary sequences—such as Alu sequences, which form intron base-pairing—were accumulated in the introns where the back-splicing events presumably occurred [18,19]. Therefore, the current model proposes that back-splicing can be caused at the proximal ends of the interacting introns of a pre-mRNA [18,19]. In addition, the interacting introns can also promote exon skipping at the distal ends [18]. This model does not contradict the hypothesis that circRNAs could be by-products of endogenous MES events.

Based on the traditional classification of alternative splicing, MES is regarded as a complex alternative splicing by sequential cassette exons [20]. One of the representative genes involved in this splicing of multiple exons is *TTN* (titin). The truncated *TTN* transcript produced by skipping was observed in the heart. It was suggested that an RNA-binding protein, RBM20, promoted intron retentions and the skipping of multiple exons via the binding to *TTN* pre-mRNAs [21]. However, it is difficult to conclude that MES with circRNA generation involves usual alternative splicing and occurs in sequential cassette exons. It is likely that most exons of circRNAs are constitutive exons rather than alternative exons [9]. It was also reported that QKI (Quaking) and MBL (muscleblind), known alternative splicing regulators, could control the biogenesis of circRNAs via changing the structures of circRNA precursors [22,23]. Analyses of *DMD* circRNAs have been performed mainly for the 5′ end of the gene [24,25]; most exons of *DMD* circRNAs are constitutive exons. Moreover, it is unclear how *DMD* circRNAs and MES events occur in the *DMD* hotspot or how they are controlled. Furthermore, the ablation of nonsense-mediated mRNA decay (NMD) produced increased aberrant splicing products, such as single exon-skipping products and MES products in mammals [26]. This was consistent with the substantial presence of circRNAs. In addition, we previously reported re-splicing of mature *TSG101* (tumor susceptibility gene 101) mRNA and *FHIT* (fragile histidine triad) mRNA [27]. Their MES products were detectable in cancer cells, and exonic lariats were produced instead of circRNAs. These results suggested that normal *TSG101* and *FHIT* mRNAs were re-spliced as pre-mRNAs.

In this study, we attempted to detect the truncated *DMD* mRNAs, especially exon 44–56-connected endogenous mRNA, from around the *DMD* hotspot. The exon 44–56-connected mRNA transcript, which has skipped all the exons of the *DMD* hotspot, should be an ideal induction target in MES therapy. Using normal human skeletal muscle total RNAs, we successfully detected the endogenous expression of this induction target (exon 44–56). In addition, this result was supported by the endogenous expression of its hypothetical by-product (the exon 55–45 *DMD* circRNA). We also observed that the 3′ end of exon 44 was more efficiently involved not only in the generation of exon 44–56, but also in other MESs around the *DMD* hotspot. Recently, Gazzoli et al. reported an initial view of post-transcriptional *DMD* pre-mRNA in muscular cells [28]. Indeed, they characterized *DMD* introns into non-sequential introns (including post-transcriptionally spliced introns:post-transcriptional introns) and sequential introns (so-called co-transcriptionally spliced introns or co-transcriptional introns). *DMD* intron 44 was annotated as a post-transcriptional intron [28]; therefore, it was reasonable to suggest that it could take part in intronic interactions with downstream introns for the MES events and the generation of circRNA, similar to the current model described above. This is the first report to suggest a relationship between MES with circRNA generation and post-transcriptional introns. We believe that these results will contribute to the development of RNA therapies, such as multiple exon-skipping therapy.

## 2. Results

### 2.1. Detection of the Endogenous Multiple Exon-Skipping Products from around the DMD Hotspot

**Figure 1:**
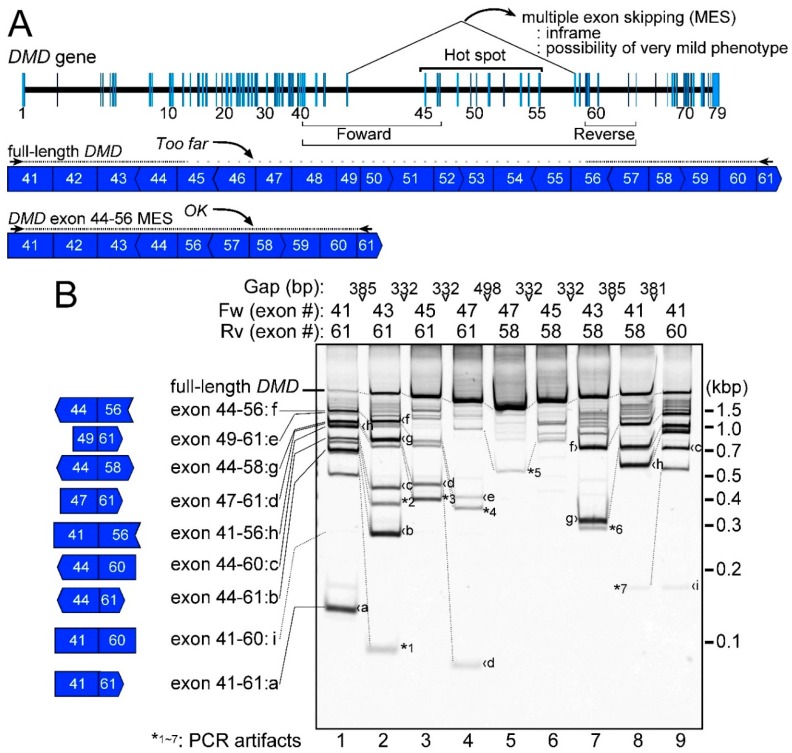
Expression analysis of multiple exon skipping (MES) products. (**A**) A schematic of the Duchenne muscular dystrophy (*DMD*) gene is shown in the upper panel. Nested reverse transcription polymerase chain reaction (RT-PCR) was performed to examine expression of the exon 44–56-connected *DMD* MES mRNA. Primers were designed upstream or downstream around the *DMD* hotspot to detect MES mRNAs. The concepts underlying the primer design are also indicated. Vertical ends of exons indicate that ends of exons and codons are matched. Convex ends indicate exons containing +1 nucleotide (nt) for codons, and concave ends indicate exons showing −1 nt; (**B**) RT-PCR results for MES are shown. Inner primers and the gaps to the next primer are indicated at the top. Molecular markers are indicated on the right side and detected MES products are shown on the left. A schematic of the products on the left side indicates the connecting exons by MES. Broken lines on the panel indicate identical products. Asterisks show PCR artifacts and are explained in Supplementary File 1.

We investigated MES mRNAs from around the *DMD* hotspot using nested reverse transcription polymerase chain reaction (RT-PCR) using human normal skeletal muscle total RNAs. The outer primers of the nested PCR were designed against exons 39 and 62, and the inner primers were designed against exons 41 and 61 (Figure 1A,B, lane 1). Although the 30 s extension time of the PCR cycles was insufficient to amplify the full-length *DMD* mRNA, we observed various shorter PCR products (Figure 1B, lane 1). When the inner primers were replaced with closer primers, the full-length products increased according to the proximity of the inner primers (Figure 1B, lanes 2–9). Although 45 PCR cycles were performed in this experiment, the density of the band corresponding to the full-length product was broadly proportional to the proximity of the primer set: the strongest band for the full-length product was gained using the closest primer set (Figure 1B, lane 5). This indicated that the PCR amplification was not in the plateau phase and could be used for quantification.

To fully detect these shorter products from around the *DMD* hotspot, primers with different annealing positions around this area were used. As the full-length product showed an altered mobility, the shorter products also changed their mobilities or disappeared, basically according to the primer positions used (Figure 1B, lanes 1–9). Various primer sets were tested in this experiment to find as many MES products as possible. Sixteen different types of shorter *DMD* products were observed (Figure 1B, products **a**–**i** and asterisks 1–7). Nine types of products were *MES* DMD products, which had gapped connections between distant but conventional splice sites (Figure 1B, products **a**–**i**). Surprisingly, product **f** was identical to the exon 44–56-connected *DMD* mRNA, which resulted in MES from exon 45 to 55, and was the induction target of the proposed multiple exon-skipping therapy (Figure 1B, lanes 1, 2, 7–9). The exon 41–60-connected MES *DMD* product (exon 41–60, product **i**), was only observed as a weak band in Figure 1B, lane 9, but not in lane 1. The remaining seven types of shorter products were regarded as PCR artifacts (Figure 1B, asterisk 1–7). The detection conditions using 45 PCR cycles could be the maximum limit of the experiment for the *DMD* hotspot. Therefore, eight MES products (except for product **i**) that we identified around the *DMD* hotspot were representative of the MESs that happened in normal human skeletal muscle.

### 2.2. CircRNA Products as Hypothetical By-Products of MES Products

**Figure 2:**
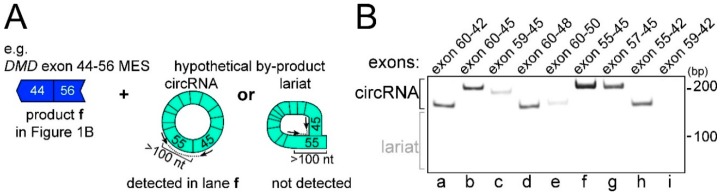
Expression analysis of circular RNA (circRNA) products. (**A**) A schematic of multiple exon skipping (MES) product and its hypothetical by-products. A case of the exon 44–56-connected *DMD* MES is shown as an example. Although circRNA is generally a candidate of the hypothetical by-product, exonic lariat is another candidate by the re-splicing model [27]. Divergent reverse-transcription polymerase chain reaction (RT-PCR) can amplify circRNA and exonic lariat; (**B**) RT-PCR results for hypothetical by-products of MESs. Exons involved in back-splicing or re-splicing are indicated at the top. Molecular markers are indicated on the right. No lariat exon-type products were detected.

Next, we attempted to confirm the expression of a hypothetical by-product for each MES product using divergent RT-PCR. PCR primers for divergent RT-PCR were designed to bind to the genome facing away from each other. Thus, these primers theoretically amplify circular-type products, but not mRNAs. It was hypothesized that a circRNA product generated by back-splicing could be produced as a by-product of MES [18,19]. Ultimately, we detected eight circRNA products that were hypothetical by-products of the MES products, except for the exon 41–60 product (Figure 2B). In other words, the exon 55–45-connected *DMD* circRNA product (exon 55–45 circRNA) was detected in normal human skeletal muscle total RNAs (Figure 2B, lane f). However, we failed to detect the exon 59–42 circRNA product, which was a hypothetical by-product of the exon 41–60 MES product (Figure 2B, lane i). As mentioned above, the exon 41–60 MES products were rarely expressed (Figure 1B). In addition, exonic lariats whose gapped sequences were connected between an exonic branch-point and the 5′ splice site were not detected. Taken together, these results suggested that the eight types of MES events with circRNAs should be considered minor splicing events compared with normal pre-mRNA splicing, but occurred frequently around the *DMD* hotspot.

### 2.3. Features of MES with CircRNA

**Figure 3:**
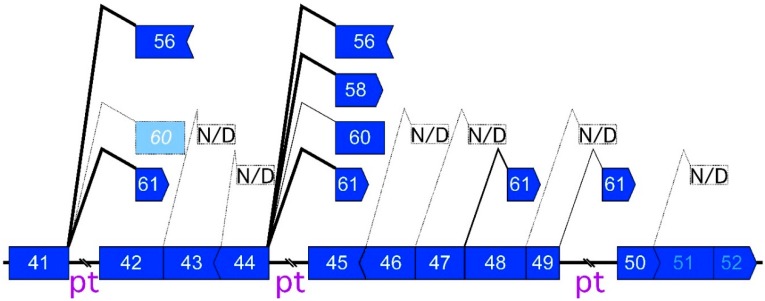
Multiple exon skipping (MES) around the *DMD* hotspot. Exon combinations of MES are shown with mountain lines on the post-transcriptional pre-mRNA, which was reported by Gazzoli et al. [28]. A broken mountain line means that a MES product was not detected (N/D), or was not validated by a hypothetical by-product, such as the exon 59–42 circRNA product (light blue). Bold lines between exons indicate post-transcriptional introns (pt), and co-transcriptional introns were eliminated between exons here. Vertical ends of blue rectangles indicate that ends of exons and codons are matched. Convex ends indicate exons containing +1 nucleotide (nt) for codons, and concave ends indicate exons showing −1 nt. The donor site of exon 51 or 52 might be too close to detect MES products that used acceptor sites around the *DMD* hotspot.

There are almost 100 combinations of exons around the *DMD* hotspot that could participate in MES if skipping of more than five consecutive exons is detectable. Despite this variety, only eight MES products were actually identified (Figure 1 and Figure 2). All eight MES products have in-frame amino acid codons, which suggested that the generation of MES products avoided NMD, similar to general mRNAs (Figure 3). However, the effect of NMD would still be insufficient to limit MES to eight combinations. There were four donor sites (the 3′ ends of exons 41, 44, 47, and 49) and four acceptor sites (the 5′ ends of exons 56, 58, 60, and 61) that participated in the eight MES events (Figure 3). Although the four acceptor sites of the MES events were all sites that form in-frame codons with exons 56–61, three hypothetical donor sites (the 3′ ends of exons 42, 46, and 48), which also form in-frame codons, were not used for the MES events. In addition, other hypothetical donor sites (3′ ends of exon 43, 45, and 50) have different hypothetical acceptor sites to form in-frame codons. However, these hypothetical sites were not used in the MES events (Figure 1 and Figure 3). Moreover, the donor site of exon 44 was a preferential site for MES with circRNA generation because it was used in four of eight combinations (Figure 3). Two donor sites of the eight combinations were located at exon 41. Therefore, we focused on the reason why only four donor sites, and especially that of exon 44, were used for the MES events.

Recently, Gazzoli et al. reported non-sequentially spliced introns and sequentially spliced introns of the *DMD* gene by capturing pre-mRNA sequencing [28]. A sequentially spliced intron could be considered as a co-transcriptionally spliced intron (co-transcriptional intron). Non-sequentially spliced introns included not only post-transcriptionally spliced introns, but also introns involved in recursive splicing and nested splicing [28,29,30]. Recursive and nested splicing produce intermediate pre-mRNAs by intra-intronic splicing. By contrast, post-transcriptional introns are thought to be excised frequently after downstream introns are transcribed. According to results of Gazzoli et al., there were three post-transcriptional introns (including intron 44) from exon 41–50. Three post-transcriptional introns were located at the donor sites of MES events (exon 41/intron 41, exon 44/intron 44, exon 49/intron 49). Except for exon 47, the other 5′ splice sites of co-transcriptional introns were not used for MES events (Figure 3). Comparison of our detected MES and their reported post-transcriptional *DMD* pre-mRNAs indicated that post-transcriptional introns play a key role in the occurrence of MES events. Meanwhile, we used bioinformatic approaches to analyze RNA sequences of splice sites and exonic sequences [31,32,33]. However, we could not find any reasonable results for the donor site or the acceptor sites using the Shapiro and Senapathy score (SSS), MaxEntScan, and ESE finder (ESE; exonic splicing enhancer). For example, the donor site of exon 44 showed a remarkably high splice-site score (SSS: 99.76, MaxEnt: 11.00), compared with the averages (SSS: 84.62 S.D. ± 3.02, MaxEnt: 8.61 S.D. ± 1.06) of exons in the hotspot (exon 45–55). This was contrary to those of exon 41, which scored low (SSS: 73.74, MaxEnt: 7.35).

### 2.4. Low Frequency of MESs with CircRNA

**Figure 4:**
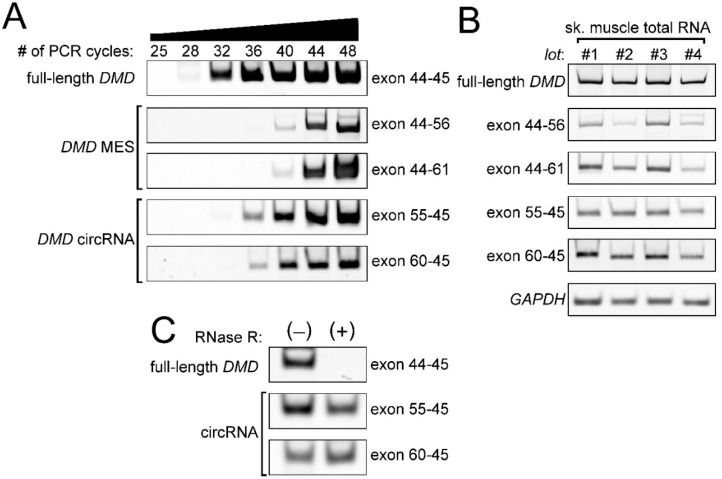
Expression of the exon 44–56-connected multiple exon skipping (MES) *DMD* mRNA. (**A**) Semiquantitative reverse transcription-polymerase chain reaction (RT-PCR) experiments for the exon 44–56 and its hypothetical by-product (exon 55–45 circRNA) were performed using human skeletal muscle total RNA. Because exon 61 frequently functioned as an acceptor of MES, the exon 44–61 and its partner (exon 60–45 circRNA) were also investigated. Total cycle numbers are shown on the upper side; (**B**) RT-PCR experiments using different total RNAs were performed. Total numbers of PCR cycles were 30 cycles for the full-length *DMD* mRNA, 42 cycles for the MES products (exon 44–56, 44–61), 37 cycles for the circRNA products (exon 55–45, 60–45), and 23 cycles for *GAPDH* (glyceraldehyde-3-phosphate dehydrogenase); (**C**) RNase R treatment for exon 55–45 *DMD* circRNA. Total RNA was treated by RNase R, followed by RT-PCR. Total PCR cycles were 30 cycles for the full-length *DMD* mRNA and 40 cycles for the circRNAs.

Most post-transcriptional introns are conventionally spliced to produce normal mRNA just like co-transcriptional introns. It is expected that the frequency of the exon 44–56 MES would be low. To confirm the low frequency of these unusual, but endogenous, activities, we performed classical (but nested) semiquantitative RT-PCR for the exon 44–56 product and its partner: the exon 55–45 circRNA product. Either exon 44 as a donor site and/or exon 61 as an acceptor site was used in seven of eight MES products; therefore, we also tested the exon 44–61 event and its partner: the exon 60–45 product. A strong signal of the full-length *DMD* product was observed in 32 cycles. Those of the exon 44–56 product and the exon 44–61 product were observed in 44 cycles. Compared with expression of the full-length *DMD* mRNA, both MES products were expressed at lower levels, with a difference of approximately 12 PCR cycles (Figure 4A). After densitometry analysis, the curves showing the increases in the amounts of the full-length mRNA and the MES products were similar and suggested a difference of approximately 1.7-fold per cycle. Therefore, a difference of 12 PCR cycles suggested that the full-length mRNA was expressed roughly 600-fold higher than the MES products. Meanwhile, both the circRNA products were also expressed at lower levels, with a difference of approximately 4–8 PCR cycles compared with the expression of the full-length *DMD* mRNA, but were expressed at higher levels than their partner MES products (Figure 4A).

Using multiple human skeletal muscle total RNAs, we confirmed the low expression of the exon 44–56 MES products and the exon 44–61 MES products (Figure 4B). The partner circRNA products were also observed in the different RNA sources (Figure 4B). In addition, we also performed an RNase R treatment to validate that these circRNA products were amplified from the circRNAs. Previously, we used in vitro experiments to show that circRNAs could avoid degradation by an exoribonuclease before cleavage by an endoribonuclease [25]. Indeed, we validated that the exon 55–45 product and the exon 60–45 product were derived from the circRNAs, but not the full-length mRNA (Figure 4C). Generally, the linear mRNAs were less stable than the circRNAs [10,19]. This corresponded to our results that the relative amounts of the circRNAs were clearly higher than those of their partner MES products (Figure 4A). If a strong regulatory site to stabilize an mRNA, such as an AU-rich element, is not located in the skipped exons of the MES products, the ratio of the expression of the full-length mRNA and the MES mRNA that share the sequence would indicate the frequency of the MES [34].

### 2.5. Preferential Exon Combinations of CircRNA Products

**Figure 5:**
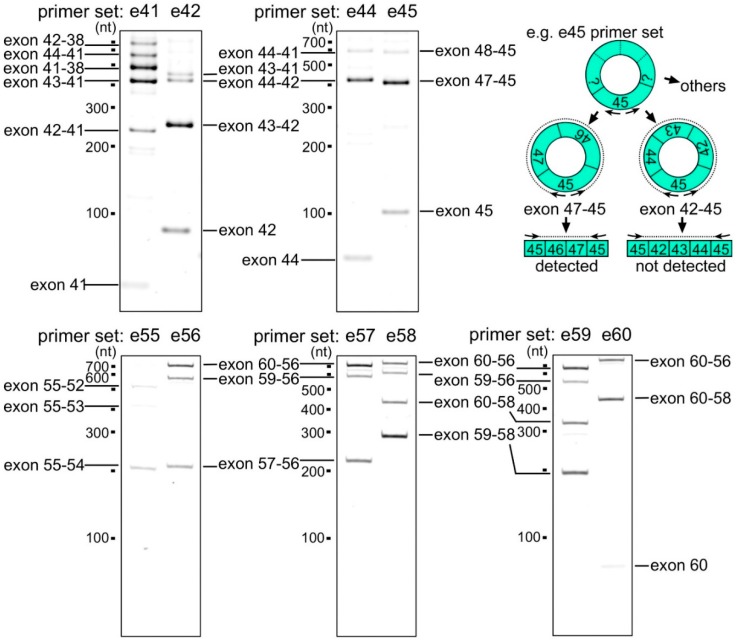
Expression analysis of small circRNAs by divergent reverse-transcription polymerase chain reaction (RT-PCR). Primer sets were designed for each exon and are indicated at the top of the panels. Molecular markers are shown on the left of each panel. The exons involved in back-splicing to generate circRNAs are indicated on either side of the panels. The upper right panel indicates that the exon 47–45 circRNA product was successfully detected using e45 primers. However, circRNA products that have both exon 44 and 45, such as the exon 45–42, were not detected by the e45 primers. Sequences of primer sets (e41, e42, e44, e45, and e55–e60) are indicated in Supplementary File 2.

In contrast to the coincidence between 5′ splice sites of post-transcriptional introns and the donor sites of the MES events, it was difficult to find any rules for acceptor sites. Only the upstream adjacent intron to exon 58 was a post-transcriptional intron, and the others were co-transcriptional introns [28]. Therefore, we focused on active sites for back-splicing. However, it was unrealistic to investigate comprehensively hundreds of possible exon combinations of back-splicing even only around the *DMD* hotspot. RNA-Seq experiments showed that frequently observed circRNAs are small and mainly span one to five exons [10]. Therefore, we designed four primers (nested primers) to perform the divergent RT-PCR on each exon (exon 40–60) as shown in Figure 5. Exon 61 was too short to design primers.

**Figure 6:**
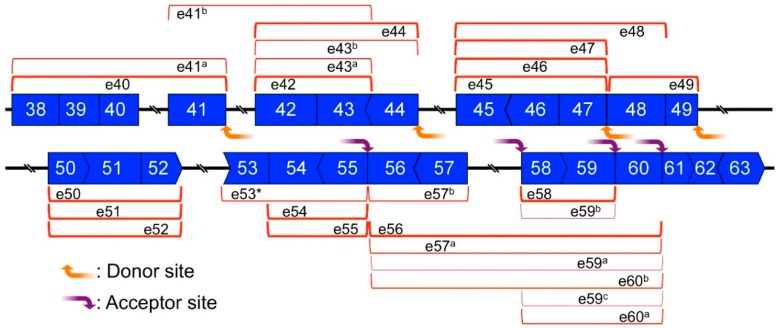
Mapping of small circRNAs. Divergent reverse transcription-polymerase chain reaction (RT-PCR) experiments were performed using skeletal muscle cDNAs and a primer set for each exon (exon 40–60). Highly expressed small circRNAs or equally highly expressing circRNAs are shown as round red brackets. Donor and acceptor sites of multiple exon skipping (MES) are indicated by orange and purple arrows, respectively. Bold lines indicate post-transcriptional introns.

First, divergent RT-PCR for exon 41, whose 3′ end was frequently used for MES, amplified several fragments (Figure 5). The exon 41–38 *DMD* circRNA product was observed frequently in human skeletal muscle total RNAs. Second, the exon 43–41 circRNA product was also detected, although it was a minor product in divergent RT-PCR for exon 42, which is adjacent to exon 41 (Figure 5). Strong expression of the exon 43–42 *DMD* circRNA product indicated that the 5′ end of exon 42 was an active site for back-splicing. Exon 41, surrounded by post-transcriptional introns (Figure 6), was joined to upstream exons (exon 38–40) or downstream exons (exon 42 and 43). In the case of exon 44, whose 3′ end was most frequently used for MES, the exon 44–42 circRNA product was a major product (Figure 5), but was detected at a lower level than the exon 43–42 product in the reaction for exon 42. In addition to exon 42 and 43, exon 44 is also a member of the preferential combination. By contrast, divergent RT-PCR for exon 45 amplified mainly the exon 47–45 circRNA product (Figure 5). Among these smaller circRNAs, no circRNA including both exon 44 and 45 was observed. This result indicated that post-transcriptional intron 44 was frequently involved in back-splicing at both ends.

At the acceptor side, primers for exon 55 amplified mainly the exon 55–54 circRNA product (Figure 5). However, primers for exon 56 amplified mainly the exon 60–56 circRNA product (Figure 5). Again, no small circRNAs containing both exon 55 and 56 were observed. This indicated that intron 55 was frequently involved in back-splicing. The donor and acceptor of the exon 44–56 MES were also frequently involved in back-splicing.

Moreover, primers for exon 57 amplified the exon 60–56 and the exon 57–56 circRNA products in roughly equal amounts. By contrast, primers for exon 58 amplified mainly the exon 59–58 product (Figure 5). In addition to the exon 59–58 circRNA product, primers for exon 59 amplified the exon 60–56 or the exon 59–56 products similarly (Figure 5). Primers for exon 60 amplified the exon 60–58 and exon 60–56 circRNA products (Figure 5). We could not detect any small circRNAs containing both exon 60 and 61. This indicated that the 5′ end of intron 60 was frequently involved in back-splicing. Furthermore, our results indicated multiple layers of affinity among exon groups of the circRNA generation. Exons 56–60 formed a group, but there were multiple affinity exon subgroups in this group.

### 2.6. Mapping of Back-Splicing, Donor, and Acceptor Sites of MES, and Post-Transcriptional Introns

To simplify the complex results of each divergent RT-PCR, highly expressed circRNA product were drawn on post-transcriptional *DMD* pre-mRNA (Figure 6). For example, the exon 44–42 circRNA-product was detected as the strongest band using the exon 44 primers (Figure 5); it was mapped and is indicated using a round red bracket in Figure 6. Vertical edges of the bracket indicate splice sites that were responsible for back-splicing (Figure 6). In this study, we called these splice sites “back-splicing sites”. Namely, “back-splicing sites” are splice sites, and are involved in not only back-splicing but also normal splicing. We could design the primers on each exon from exon 40 to 60; therefore, each highly expressed circRNA product(s) was identified and indicated in Figure 6. Likewise, minimal and preferential exon combinations for circRNA were visualized on the pre-mRNA. The accumulation of vertical edges indicates frequent back-splicing sites (Figure 6).

The splice sites of all post-transcriptional introns (introns 40, 41, 44, 49, 52, and 57) were matched with detected back-splicing sites (Figure 6). Meanwhile, the back-splicing sites were observed at each donor site of the MES (introns 41, 44, and 49), where post-transcriptional sites were located. As described above, the 3′ end of exon 47 was an exception, in that a co-transcriptional site was used as the donor site of MES. Back-splicing sites were observed at intron 47, which was adjacent to the 3′ end of exon 47 (Figure 6). Similarly, frequent back-splicing sites were observed in introns that were adjacent to all acceptor sites of MES events (Figure 6). This suggested that investigating the expression of small circRNAs could help to predict MES events.

## 3. Discussion

Despite many possible combinations, the exon 44–56 MES products of the *DMD* gene were identified and validated by a hypothetical by-product: the exon 55–45 circRNA product. This MES product could represent the ideal induction target of the MES therapy [7]. MES therapy will require the artificial enhancement of this splicing between exon 44 and 56; therefore, it is important to determine its mechanism. However, it is difficult to set up artificial assay systems for MES studies because the length of the total intronic regions is too large to make a DNA construct. It is impossible to evaluate whether the assay system would follow the endogenous MES events. In this study, we attempted to infer the mechanism by identifying in vivo-produced mRNA species from the *DMD* gene.

We identified and validated eight types of MES products (Figure 1 and Figure 2). Various types of primers were used to detect as many MES products as possible. Some PCR artifacts were amplified using nested RT-PCR (Figure 1). The sequence of the hotspot includes spectrin-like repeats that might affect the amplification of PCR artifacts. In addition, the relative amounts of MES products were extremely low compared with the normal *DMD* mRNA transcript (Figure 4). Therefore, the eight types of products can be considered as representative MES mRNAs from around the *DMD* hotspot. Of course, there is the possibility that we failed to detect some MES mRNAs because it was theoretically difficult to detect single exon skipping or short MESs.

Significantly, all eight MES products had in-frame sequences, suggesting the avoidance of NMD. Generally, NMD degrades out-of-frame linear transcripts. Therefore, it was likely that out-of-frame MES products were eliminated by NMD. This elimination might have resulted in only eight MES products being detected from around the hotspot. Among the eight representative products, we found that only four donor sites (from exons 41, 44, 47, and 49) were used for the MES events. Because four of the MES products started skipping at exon 45, exon 44 is very important as the donor site of MES. In addition, two types of MES products used exon 41 as the donor site (Figure 3). However, bioinformatics analyses of RNA sequences of splice sites or exonic sequences did not reveal any significance in the donor sites or the acceptor sites. Except for the detected acceptor sites (exons 56, 58, 60, and 61), there was no in-frame exon in this area.

**Figure 7:**
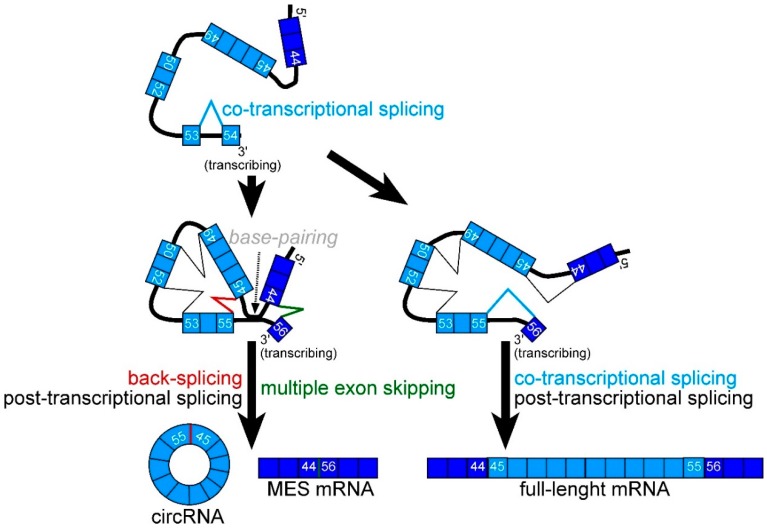
Potential flow of the exon 44–56-connected MES *DMD* mRNA and the exon 55–45 *DMD* circRNA. Our results and the previously reported post-transcriptional *DMD* pre-mRNA were overlaid onto the current model of circRNA generation in left side. Light blue lines indicate co-transcriptional splicing. Red and green indicate back-splicing and MES, respectively. Co-transcriptional introns are not shown, except for intron 53 in the upper image and intron 55 in the middle image. It was assumed that post-transcriptional intron 44 interacts with the transcribing co-transcriptional intron 55 in left side, but not in right side, for the full-length mRNA.

Interesting information was obtained from the comparison between the donor sites and the 5′ end of the post-transcriptional introns, as reported by Gazzoli et al. [28]. There are three post-transcriptional introns in the region of the donor sites of MES. The 5′ ends of these post-transcriptional introns were used as the splice sites for MES (intron 41, 44, and 49, Figure 3). By contrast, the 5′ ends of the co-transcriptional introns in this area were not used as donor sites of MES, except for the exon 47–61 MES product (Figure 3). A recently hypothesized model of the circRNA generation requires intra-intronic base-pairing to promote back-splicing. To make the introns interact, the upstream intron must not be excised before the transcription of the downstream intron(s) (Figure 7). Transcribing either the downstream intron or the post-transcriptional intron could be acceptable to allow an interaction with the upstream post-transcriptional intron. Combining our observations and Gazzoli’s results suggested that post-transcriptional introns trigger MES with circRNA generation as a donor site. Our results represent new evidence of the importance of temporary intron retention from the different viewpoint of MES with circRNA generation.

Meanwhile, we did not investigate whether post-transcriptional introns could really interact with extremely distant downstream introns. The distances between the introns next to the donor sites are at least >16,000 nt, which seems to be sufficiently long to interact with downstream introns if these introns exist in pre-mRNA. There are large numbers of repetitive sequences, including Alu, in this area. Therefore, we could not predict the intra-intronic base-pairing. In addition, our results did not conflict with an older model that suggested circRNA was formed via a large lariat intron containing skipped exons because unspliced 5′ splice sites of post-transcriptional introns might be advantageous to a splicing process against extremely distal 3′ splice sites such as MES. It is thought that lariat introns suffer debranching and rapid RNA degradation.

Each of the eight detected MES products were validated by the presence of their respective circRNA product as a hypothetical by-product (Figure 1 and Figure 2); therefore, it was obvious that the back-splicing sites were located at the ends of the adjacent intron next to each donor site or each acceptor site of the MES events. However, it is difficult to rank the activity of back-splicing sites hierarchically because there are too many combinations of exons to test comprehensively. Therefore, we performed divergent RT-PCR using primers designed for each exon and checked for highly expressed circRNA products. Each identified product represented the minimum and preferential exon combination that was involved in each exon. The accumulation of highly expressed small circRNAs is presumed to indicate the presence of active back-splicing sites (Figure 6). Although small circRNAs and large circRNAs that were hypothetical by-products of an MES product might be generated by different machineries, back-splicing sites by small circRNA products were located not only at the ends of adjacent introns next to the donor sites, but also next to the acceptor sites of the MES (Figure 6). This suggested that frequent back-splicing sites, indicated by highly expressed small circRNAs, indicated potential acceptor sites of MES events. Some frequent back-splicing sites, such as the 5′ end of exon 56, act as acceptor sites of MES events.

Our results suggested that post-transcriptional introns triggered the occurrence of MES events. The exception was the donor site exon 47. Although intron 47 was categorized as a co-transcriptional intron, this does not mean that intron 47 in all *DMD* transcripts is co-transcriptionally excised [28]. It is possible that a small number of post-transcriptional *DMD* pre-mRNAs contain intron 47. Meanwhile, the structure of the post-transcriptional *DMD* pre-mRNA was revealed using muscle cells, although we performed the detection of MES products using skeletal muscle total RNAs. This small difference of RNA sources might have influenced the results. Nucleic RNAs from human skeletal muscle were not available; therefore, we could not test post-transcriptional *DMD* pre-mRNA. In addition to intron 47, introns 53 and 55 were frequent back-splicing sites that were indicated by the presence of circRNA products and have been categorized as co-transcriptional introns. Based on the current model of the circRNA generation, these introns should have been temporally retained in the post-transcriptional *DMD* pre-mRNA. In addition, not all intron 53- or 55-containing *DMD* transcripts were co-transcriptionally excised, and the difference in RNA source might have influenced the results.

Taken together, our results suggested that post-transcriptional introns, such as intron 44, triggered the occurrence of MES events, and that frequent back-splicing sites, such as the border of intron 55/exon 56, functioned as acceptor sites of MES events. Perhaps RNA degradation by NMD controls MES mRNAs, leaving in-frame MES mRNA such as the exon 44–56-connected MES *DMD* mRNA. In addition to the reported structure of the post-transcriptional *DMD* pre-mRNA, highly expressed *DMD* circRNAs should be useful to identify donor and acceptor sites of MES events. We expect that our observations will contribute to developing artificial induction of the exon 44–56-connected MES *DMD* mRNA for MES therapy.

## 4. Materials and Methods

### 4.1. Total RNAs and cDNA Synthesis

Human normal skeletal muscle total RNAs were purchased from commercial suppliers (Ambion, Waltham, MA, USA; Clontech, Kusatsu, Japan). cDNAs were synthesized using Superscript III with random primers (Invitrogen, Waltham, MA, USA). Reaction mixtures, which were essentially prepared according to the supplier’s instruction, contained 4 µg of total RNA and 100 ng of random primer in 20 µL. The cDNA synthesis reactions comprised incubation for 30 min at 50 °C, followed by additional incubation for 15 min at 55 °C. After RNase H treatment, reaction mixtures were used as cDNA samples.

### 4.2. RNase R Treatment

The RNase R treatment was performed essentially as described in a previous report [25]. The purified RNase R enzyme and RNase R buffer were obtained from Epicentre (Epicentre, San Diego, CA, USA). The reaction mixtures for the RNase R treatment contained 4 µg of human skeletal muscle total RNA with or without 40 units of RNase R in 40 µL solutions. The incubation for RNA digestion was performed for 30 min at 37 °C. The samples were subjected to phenol/chloroform extraction, followed by ethanol precipitation. After dissolving the precipitates in water, the nondigested human skeletal muscle total RNA (4 µg) and the RNase R-digested RNA from the same source (4 µg) were used for cDNA synthesis as described above.

### 4.3. Nested PCR and Divergent PCR

The cDNAs prepared above were used for PCR experiments. Except for *GAPDH*, all PCR experiments were performed as nested PCR, comprising 1st and 2nd PCR reactions, using GoTaq Flexi DNA polymerase (Promega, Fitchburg, WI, USA). PCR reaction mixtures contained 1× reaction buffer, 2.5 mM MgCl_2_, 200 µM dNTPs, 2 µM specific primers, 0.9375 unit GoTaq DNA polymerase, and 1 µL of cDNA sample in 25 µL. PCR cycles comprised denaturing for 30 s at 94 °C, then annealing for 30 s at 60 °C, and extension for 30 s at 72 °C. After 25 cycles of the 1st PCR reactions, samples were applied to MicroSpin S-300 HR Columns (GE Healthcare, Little Chalfont, UK).

Divergent PCR to detect circRNA product was performed similarly to the nested PCR. Each primer set was designed in opposite directions against the genome sequence to amplify products that form circRNA and lariat RNA. Reaction mixtures were the same as those described above. In case of divergent PCR to detect small circRNA product, primer sets were designed in each exon. Because the time of extension in the PCR reaction is 30 s, GoTaq polymerase efficiently amplifies smaller products (approximately <1000 nt).

After application to the MicroSpin S-300 HR Columns, 2 µL of the flow-through samples were used as the template for the 2nd PCR; all other reagents were the same as the 1st PCR. The thermal cycling conditions were the same as the 1st PCR.

Essentially, 20 cycles for the 2nd PCR reaction were performed to detect MES mRNAs and 15 cycles were used to detect circRNAs. In the case of the classical semiquantitative RT-PCR, the total numbers of PCR cycles, consisting of a 1st PCR (25 cycles) and 2nd PCR, are indicated in the appropriate figure. In the case of *GAPDH*, the same concentration as that in the 1st PCR reaction was used and 23 cycles were performed [35]. PCR primers for the *DMD* gene were designed using Primer3 [36,37], and are shown in Supplementary File 2. All experiments were performed at least three times independently.

### 4.4. Experimental and Bioinformatic Analyses of PCR Products

After completion of the PCR experiments, 4 µL of samples were analyzed by electrophoresis through 6% native polyacrylamide gels. Gels were stained with SYBR green I DNA staining solution (Takara, Kusatsu, Japan). Gel images were obtained using a LAS-3000 imager (GE Healthcare). The densitometric analyses were performed using Image Gauge (GE Healthcare). Each separate band from the PCR reaction mixture was isolated and extracted from the gels. Sequences of the PCR products were determined using an ABI 3130 sequencer (Thermo Fisher Scientific, Waltham, MA, USA). The obtained sequences were compared with the *DMD* mRNA sequence and the genomic sequence of *DMD* gene, occasionally using UCSC human BLAT [38]. Shapiro and Senapathy scores and MaxEnt scores for splice sites were calculated in the splice-site tool and using MaxEntScan [31,32]. ESE scores were also examined using ESE finder [33].

## 5. Conclusions

We observed low expression of eight kinds of MES products of *DMD* mRNAs, which includes the exon 44–56-connected MES product: an ideal induction target for MES therapy. A comparison between our observations and Gazzoli’s recent report suggested that post-transcriptional introns, such as intron 44, trigger the occurrence of MES events. It is reasonable that temporal retention of upstream post-transcriptional introns assists in the interaction with the post- or co-transcriptionally spliced downstream introns. The back-splicing sites, indicated by the presence of highly expressed small circRNA products, were found in all donor and acceptor sites of MES events. Thus, investigation of circRNAs could identify poorly expressed MES mRNAs. These findings will contribute to the artificial and specific enhancement of the exon 44–56-connected MES *DMD* mRNA, which has the possibility to cure DMD patients.

## Supplementary Materials

Supplementary materials can be found at www.mdpi.com/1422-0067/17/10/1722/s1. Supplementary File 1: Explanation of PCR artifacts in Figure 1B; Supplementary File 2: Sequences of PCR primers. Sequences of all PCR primers are shown. Outer primers were used in the 1st PCR and inner primers were used in the 2nd PCR.

## Abbreviations

BMD	Becker muscular dystrophy
*CDR1as*	Complementarity Determining Region1 antisense
circRNA	circular RNA
DMD	Duchenne muscular dystrophy
EIcircRNA	Exon-intron circular RNA
ESE	Exonic splicing enhancer
*FHIT*	Fragile Histidine Triad
MaxEntScan	Maximum Entropy Scan
MBL	Muscleblind
MES	Multiple exon skipping
NMD	nonsense-mediated mRNA decay
QKI	Quaking
RT-PCR	reverse transcription and polymerase chain reaction
SSS	Shapiro & Senapathy Score
*TSG101*	Tumor susceptibility gene 101
*TTN*	titin
