# Supplementary material for: Endogenous Multiple Exon Skipping and Back-Splicing at the DMD Mutation Hotspot
Source: Int J Mol Sci. 2016 Oct 13;17(10):1722. doi: 10.3390/ijms17101722 (PMC5085753; doi:10.3390/ijms17101722)
Supplement: Supplementary file 1 [file ijms-17-01722-s001.zip › Supplementary file 1.pdf]

Supplementary Materials: Endogenous Multiple Exon Skipping and Back-Splicing at the *DMD* Mutation Hotspot

Hitoshi Suzuki, Yoshitsugu Aoki, Toshiki Kameyama, Takashi Saito, Satoru Masuda, Jun Tanihata, Tetsuya Nagata, Akila Mayeda, Shin'ichi Takeda and Toshifumi Tsukahara

When we investigated multiexon-skipping (MES) mRNAs from around the *DMD* hotspot using nested reverse transcription polymerase chain reaction (RT-PCR) using human normal skeletal muscle total RNAs, sixteen different types of shorter *DMD* products were identified (Figure 1B, products **a**–**i** and asterisks 1–7). As we described in the main text, nine types of products were *MES* DMD products, the remaining seven types of shorter products were regarded as PCR artifacts because their sequences contained overlapping sites (>6 nt) or had primer mis-annealing sites (Figure 1B, asterisks 1–7). These sequences could not be explained using the consensus sequence of pre-mRNA splicing. The appearance of artifacts indicated that the detection conditions using 45 PCR cycles could be the maximum limit of the experiment for the *DMD* hotspot. These artifacts also changed their mobilities according to the primer positions (Figure 1B). Including the MES products, 16 types of products covered most of the short *DMD* products that appeared using the most distant primer set (Figure 1B, lane 1). Moreover, the closest primer set amplified mainly an artifact and the full-length product (Figure 1B, asterisk 5). Therefore, these identified MES products around the *DMD* hotspot were representative of the MESs that happened in normal human skeletal muscle.
